# Acetaldehyde mediates the ethanol effects in developing brain

**DOI:** 10.3389/fnbeh.2013.00075

**Published:** 2013-07-01

**Authors:** S. M. Zimatkin

**Affiliations:** Department of Histology, Cytology and Embryology, Grodno State Medical UniversityGrodno, Belarus

The mini review of March et al. (2013) is devoted to a very interesting problem: the role of acetaldehyde in the effects of ethanol in early postnatal development. It summarizes and analyzes carefully the literature available on the problem (about 120 references in the list) and in general the review looks quite comprehensive.

In the beginning, as an introduction, the authors provide epidemiological data concerning the early initiation of alcohol consumption in children. In addition they mentioned the possibility of involuntary alcohol exposure of fetus or newborn as a result of maternal alcohol consumption during pregnancy and breastfeeding. It can induce both a dramatic disturbances in all organs, especially in the brain, and increased alcohol consumption in their future life. It may be postulated that “the earlier beginning of alcohol exposure, the higher risk of alcoholism development.” It confirms the need of using the animal models to understand the mechanisms of early alcohol effects.

Then the authors describe the data available on the pharmacological, mostly behavioral, effects of ethanol in early ontogeny. They indicate a very intensive consumption and no aversion to high concentrations (doses) of ethanol in naïve infant rats, in contrast to adult ones. The neurochemical mechanisms of those specificities of alcohol related behavior is still unclear.

The next section of the review is called “The acetaldehyde hypothesis.” The authors speculate there that early ethanol acceptance may be due to high acetaldehyde (AC) generation from ethanol in the brain, along with low AC generation in the periphery. Indeed, the high central AC generation can be due to the increased activity in pups brains of catalase, the main ethanol-oxidizing enzyme in the brain of adults. Unfortunately, nobody measures in pups another important enzyme, Cytochrome P450 2E1, also oxidizing ethanol in adult brains (Zimatkin et al., 2006).

Another side of the process is the removal of ethanol-derives AC by aldehyde dehydrogenase (ALDH). Therefore, it is better to talk about the accumulation of AC as a result of the balance between AC production and elimination. The accumulation of ethanol-derived AC in the brain structures in early ontogeny can be more significant then in adults due not only to its higher generation, but also its insufficient removal, because of the known lower activity of ALDH. It was found that ALDH activity (with acetaldehyde as a substrate) in the rat brain neurons of various types in fetus was very low and up to the 10th postnatal day makes 45–70% of adult level, and then increases sharply, and by the 20th day approaches the definitive level (Zimatkin and Lis, 1990). Both the higher catalase and low ALDH activity provide the conditions for higher accumulation of AC in the brain. But nobody measures the level of ethanol-derived AC in pups *in vivo*.

In addition, the induction of catalase following intragastric administration of ethanol to pups on the 5th and 6th day after birth as well as appearance of AC adducts with proteins in the brain have been found (Hamby-Mason et al., 1997). Following intragastric administration of ethanol to rats during pregnancy we found the increased activity of brain ALDH in pups. It can impact to accumulation of ethanol-derived AC in the developing brain.

Another problem is the possibilities for production and elimination of AC in the specific brain structures and types of neurons, responsible for the special brain functions, like dopaminergic neurons (with specifically high activity of catalase and low activity of ALDH) (Zimatkin and Lindros, 1996), activation of which as known mediates the reinforcing properties of ethanol.

The final section of the review is devoted to the behavioral effects of acetaldehyde during early ontogeny. The important role of AC in mediation of the motivational and motor effects of ethanol has been demonstrated. Blocking of AC formation from ethanol (by catalase inhibitors) or sequestering of central AC (by d-penicillamine) decreases the ethanol behavioral effects and stimulation of mesolimbic dopamine system directly of through the opioid system. The provided meta analysis of the literature data demonstrates a high positive correlation between brain catalase activity and ethanol consumption in developing rats, demonstrating the important role of ethanol-derived AC in alcohol craving in early ontogeny.
